# Early Palliative Care Team's Perception of Relational Work for Advanced Cancer Patients: A Qualitative Study

**DOI:** 10.1002/pon.70283

**Published:** 2025-09-19

**Authors:** C. Bouleuc, L. Thery, E. Gilbert, A. Burnod, C. Laouisset, T. Marchal, E. Legrand, J. C. Mino

**Affiliations:** ^1^ Supportive and Palliative Care Department Institut Curie Paris France; ^2^ University Paris Cité Paris France; ^3^ Université Paris‐Saclay Centre de Recherche en Epidémiologie et Santé des Populations (CESP) Inserm Villejuif France; ^4^ Oncology Department Centre Lacassagne Nice France; ^5^ Le Havre University Le Havre France; ^6^ SHARE Research Unit Institut Curie Paris France

**Keywords:** advance care planning, advanced cancer, communication, end‐of‐life care, end‐of‐life discussion, end‐of‐life trajectory, palliative care, qualitative research, serious illness conversation

## Abstract

**Background:**

In a day hospital stay, the palliative care (PC) delivery includes multi‐disciplinary assessment of palliative care (PC) needs, with treating oncologist and supportive care professionals with a long time allocated for discussions with the patients and family. But little is known about the specificity of the relational work delivered by the PC team.

**Aims:**

The aim of this study was to explore the nature of the relational work carried out at the PC day hospital from the perspective of the PC team.

**Methods:**

This qualitative research used semi‐structured open‐ended interviews, conducted and analysed according to grounded theory. Twelve physicians and twelve nurses from PC team were interviewed over a period of 5 months to collect clinical cases.

**Results:**

The analysis resulted in three main categories that explain the processes of relational work during PC Day Hospital sessions: (1) Patient engagement is necessary to increase relational work and gain patients' confidence. This involves initiating medical exchanges from the patient's point of view to obtain their participation; (2) Raising awareness of irreversible decline and approaching death using interactive relational work tactics, in line with oncologists when making decisions and announcing the discontinuation of cancer treatment; (3) Managing the final phase of the cancer trajectory requires negotiating home care terms and being aware of the family's influence.

**Conclusions:**

PC day hospital in cancer centres allows a pluri‐professional relational work including the oncologists which facilitates serious illness conversation, advance care planning and end‐of‐life care management for advanced cancer patients.

## 1 Introduction

Randomized studies and meta‐analyses showed clinical benefits of integrated palliative care (PC) in oncology settings, enhancing patients' and caregivers' quality of life, decreasing symptoms intensity and aggressiveness of end‐of‐life (EOL) care [1, 2, 3, 4, 5, 6, 7]. The American Society of Clinical Oncology (ASCO) guidelines about the integration of PC into standard oncology care, recommend systematic PC interventions carried out by multidisciplinary PC teams for patients with advanced cancer [8]. More recently, the concept of team‐based, timely, and targeted palliative care was developed in response to the need for patient‐centred symptom management, including preventive interventions to minimise crises at the end of life [9, 10]. Various referral criteria have been described, mainly severe symptoms and distress, poor prognosis, patients or family request or disease‐related criteria [11, 12, 13]. The core components of PC interventions have been described for adult cancer patients: holistic assessment, discussion of illness' prognosis and goals of treatments, support and reduction of suffering, advance care planning and EOL care [14, 15].

Previous studies have described the experiences of patients and caregivers when receiving early PC. Those experiencing symptoms particularly valued prompt attention to their physical concerns, whereas those not experiencing symptoms valued guidance in medical decision‐making and preparation for the future [16, 17]. From patients and relatives “point‐of‐view,” the main principles of relational work are that care should be flexible, attentive, patient‐led and family‐centred [18]. Other studies have used external observations to analyse the content of PC work. Initial contacts focussed on building relationships and rapport with patients and their families. Subsequent contacts focussed on understanding the illness, raising awareness of the prognosis and discussing hospice care and advance care planning [14, 19]. Another ethnographic study based on 242 h of participant observation revealed that the PC team's main message was that patients and their relative's matter [20]. However, little research has been conducted into the views of PC health professionals regarding their communication strategy.

PC interventions require outpatient consultation facilities [15]. We have already described the functioning of the PC day hospital integrated into a cancer centre [21]. During 2–6 h duration of a day hospital stay, the PC delivery includes: assessment of palliative care needs, intervention of the treating oncologist and required supportive care professionals (psychologists or psychiatrists, social workers, physiotherapists, or dieticians), adapted medical investigations and technical care, with a long time allocated for discussions with the patients and family. The PC day hospital should be distinguished from an integrative cancer centre, which focus on complementary medicine and from hospice day care units, which focus on occupational activities, rehabilitation and psychosocial support. PC day hospital is a clinical environment that focuses on providing palliative care for advanced cancer patients in the declining disease phase [22]. The aim of this study was to explore the nature of the relational work carried out at the PC day hospital from the perspective of the PC team. In this qualitative study, we conducted semi‐structured interviews with the PC specialist team and applied a data‐driven, inductive content analysis.

## 2 Methods

### 2.1 Setting

This qualitative study took place in two comprehensive cancer centres, Institut Curie at Paris and Institut Lacassagne at Nice. In both cancer centre, the PC Day hospital organisation are similar and have already been described [21]. Patients referred to the PC day hospital are in the last months before death, living at home with symptoms and reduced autonomy, facing the transitional phase when anti‐cancer treatments efficacy is exhausting, and the likelihood of dying is approaching [23]. Stays in PC day hospital last several hours and regular appointments are scheduled, a few weeks apart as needed, most often monthly.

This qualitative study is an ancillary part of a multicentre randomised trial (NCT04604873) aiming to compare quality of life and the aggressiveness of EOL care between PC day hospital and conventional PC clinics, which has been previously described, and the results of which are currently being analysed [24].

### 2.2 Design

We conducted a qualitative study with PC nurses and physicians working in the PC day hospital using a grounded theory approach [25]. We used a grounded theory approach as we were focussed on identifying key features of the PC team's practices that help to explain how they try to support patients.

The Standards for Reporting Qualitative Research (SRQR) were referred to in the reporting of this study [26].

### 2.3 Data Collection

All PC health professionals working in PC day hospital were eligible These one‐on‐one interviews were conducted in person and lasted between 45 and 90 min. The interview focussed on the clinical case chosen by the health care professional among the patients managed the day before. These patients were female in 22/38 cases, were 19–86 years old and had a variety of primary cancers (breast, lung, prostate, gynaecological, digestive cancers as well as sarcoma and melanoma). A guide was used to collect the patient's trajectory and illness management: what was the situation and the problems? What did PCday hospital professionals do precisely? What were their interactions with patients and family? What did they explain to them? What were their objectives and what did they think about the situation? Interviews were audio‐recorded with the participants' consent, the verbatim was transcribed and anonymised.

To avoid the risk of contamination, the researchers adopted a neutral attitude during the interviews, and no discussion about the content of the interviews took place between the PC healthcare professionals during the entire study period.

### 2.4 Data Analysis

The two field researchers were a female PhD sociologist and a male MD PhD health service researcher and sociologist, with wide experience of research on palliative care facilities over 20 years using interviews and observational qualitative methods.

This qualitative research was based on grounded theory, which uses an inductive analysis of data collection to highlight the social processes underlying the trajectories and practices of healthcare professionals. The researchers drew on the theoretical perspective opened by A. Strauss in his research on death and dying as a temporal process, and his approach in terms of illness trajectory and different types of medical works [27, 28, 29, 30]. Treating the patient with humanity in a caring approach is essential in palliative care ethics. However, we examined relationship with patients not from an ethical viewpoint, but as a part of the management of the EOL trajectory.

Using the grounded theory method, the researchers analysed data collected to build concepts and categories, and to identify key processes. After each interview, they began by analysing the transcripts separately, reading each carefully, coding, and noting questions and ideas in a research diary. The researchers then organised meetings together each month to stimulate reflection. In a first step the researchers worked separately to produce an inductive description of the PC day hospital functioning. In a second step, each researcher read the other's material for triangulation purposes, to compare the descriptions, and check there were only minor differences linked to institutional and organisational contexts. In a third step, they produce a common analytic description of the relational work in PC day hospital. Thus, they discussed the results with the PC professionals interviewed who reread the draughts.

## 3 Results

**FIGURE 1:**
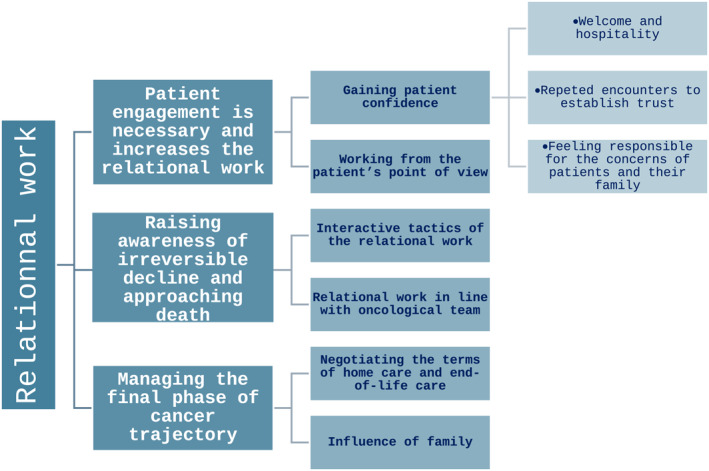
Main categories found in the qualitative analyses according to grounded theory.

From September to December 2020, 38 interviews were conducted with 6 nurses and 6 physicians (3 or 4 interviews for each physician and nurse). All PC health professionals of the two centres accepted to be interviewed. There were 2 males and 10 females, their age ranged between 32 and 60 years, and there were specialised in PC and had worked in the PC Day hospital for two years at least. We report on the main categories which explain the processes of relational work during sessions in the day hospital PC unit (Figure 1).

## 4 Patient Participation is Necessary for Relational Work, Which in Return Increases It

Relational work in PC day hospital is made possible because of patient involvement which is in turn made possible by some essential principles.

### 4.1 Gaining Patient Confidence

#### 4.1.1 Welcome and Hospitality

Hospitality helps gain the patient's trust and involvement. This is signalled through gestures or small symbolic signs, with the nurses playing a major role. This is made possible by the fact that PC team can organise itself on its own premises and see the patient for a longer period than in outpatient or inpatient consultations. Some patients reported that they had never been given such consideration before in hospital.

It creates a special atmosphere, they feel welcomed, that is very important.

(Nurse 1)

The nurse takes good care of them, some patients have their habits, the hot drink, the cake, they are made comfortable and confident.

(Physician 2)

#### 4.1.2 Repeated Encounters to Establish Trust

Before the first meeting in the PC day hospital, the patient and his treating oncologist have established for many years a trusting relationship. To gain patient confidence, several visits to the PC day hospital are needed. The patient's perception of actual support, as well as the feeling of security provided by the availability of the PC team, always reachable in the working hours, also contributes to gaining patient confidence.

It’s a good thing when they draw a benefit, that is to say when they feel better after one or two day hospital stays.

(Nurse 1)

Progressively, the PC physician acquires a central role in treating the symptoms.

(Physician 6)

#### 4.1.3 Feeling Responsible for the Concerns of Patients and Their Family

At PC day hospital, the patient's complaints and needs are the unconditional focus of interest and trigger of any medical intervention. This sometimes modifies the trajectory according to patients' request. When delivering medical information, PC professionals make special efforts to ensure clarity and education, using common words and explaining medical terms. In addition, they consider it is their prerogative and not only that of psychologists, to encourage the patient's expression of emotions or sadness.

You must adjust to the patient, look for what is bothering him, what is making him suffer, what complicates his life.

(Nurse 6)

What matters is the hierarchical order of bothers for the patient.

(Physician 1)

With this patient I was in trouble because of his low platelet rate; but for him the problem was the wet urinary probe and that he pisses on him.

(Physician 1)

I like to be alone with patients and their loved ones, explaining terms they have trouble understanding.

(Nurse 4)

### 4.2 Working From the Patient's Point of View

The PC team has adopted an essential rule: starting from patient's point of view and his/her knowledge of the illness. This means not to imposing anything in the choice of themes discussed, nor in the decisions on treatment and care, but negotiating them openly. Nevertheless, PC professionals need to some extent to guide the patient, starting from his level of bad prognosis awareness, and helping him to become more conscious of the incurability and thus of the untreatable nature of the disease.

We adopt a creative approach with the patient, and he knows that we will not impose anything.

(Physician 6)

He (the patient) accepts us, we will guide him.

(Physician 5)

Making him go through things he would never have thought of, that’s the hardest thing.

(Physician 5)

## 5 Raising Awareness of the Irreversible Decline and Approaching Death

### 5.1 Interactive Tactics of Relational Work

The PC team at the day hospital need to talk about disease progression and its evolving risks: worsening physical condition, the untreatable nature of the disease, poor prognosis and short‐term life expectation, the approaching end of life (EOL). Leading these sensitive discussions is a challenging step as patients are increasingly worried about their worsening clinical condition. It is important to employ interactive tactics and to avoid brutal announcement or confusing information with oncologists.

It is a conversation with open questions to help the patient talk (…) we need him to verbalize his feelings, we avoid closed questions.

(Nurse 4)

We use rewording techniques, and if the answers are evasive, we probe for more.

(Nurse 4)

I try to test the water and get the patient talking: what did your oncologist say? What do you think about it? Or else I leave a door open.

(Physician 5)

The interview goes crescendo, at the start we are on symptoms, then gradually (…) the discussion goes deeper.

(Physician 5)

If we talked about sensitive issues last time, I come back to it, I say « remember what we said the last time, do you want to take it up again? » It’s just so they can talk, whether they take it or not.

(Physician 6)

### 5.2 Relational Work in Line With the Oncologist

Usually, oncologists are in favour of avoiding a missed chances for the patient, while PC teams are in favour of discontinuing to facilitate the preparation for death. At PC day hospital PC team and treating oncologist discuss together to agree on the best decision for the patient.

It (PC day hospital) is more comfortable for the oncologist than a 15‐minute slot running an hour late. Here there is a framework, the patient is in a bed.

(Physician 4)

I need to hear (what the treating oncologist thinks) to know what I can say and to feel legitimate in saying so.

(Physician 4)

It is important not to break the alliance with the oncologist.

(Physician 1)

The PC physician and the oncologist must agree on the communication strategy.

(Physician 1)

## 6 Managing the Final Phase of the Cancer Trajectory

### 6.1 Negotiating the Terms of Home Care and End‐Of‐Life Care

Negotiation is often needed to get the patient to accept home care (such as morphine, medical bed, hospice care) because of his reluctance or denial to accept the reality EOL. Every Healthcare professional, PC team or supportive care professionals (social worker, psychologist, physiotherapist…) can interact with the patient and family and then consult each other outside the bedroom to discuss the ways of improving care management or obtaining patient consent.

We say to the patient: « We have an idea of what would be better for you today for your symptoms, we are going to discuss it and then you will decide for yourself». It is wise not to rush them: because then, when they have trust and when we say « now we really need to set up morphine» (…) they know that it is really respectful of their wishes (…).

(Physician 6)

We find common ground on what they want or accept, and we change the cursor as we go along.

(Physician 6)

Introducing palliative care is a first victory, talking about the palliative care teams a second victory, and considering hospice care a third victory.

(Physician 5)

Preparing an early application for admission to hospice care, which will be activated In case of aggravation, avoids consultations or hospitalisation in emergency.

(Nurse 2)

### 6.2 Influence of the Family

The family also participates in the discussions and can help or slow the patient's awareness of the incurable and untreatable status of his disease, as well as the establishment of the EOL care plan. In the PC approach, relatives have the particularity of being both accompanying and accompanied. Family members have a major impact on the feasibility of a home stay and home death, and PC professionals contribute greatly to their support, for instance by offering them individual encounters.

She (the patient’s wife) was in total distress, so she no longer felt able to manage her husband at home. This patient (..) was very frail, but I think there was nothing that could not have been managed at home, no refractory symptoms or things like that.

(Physician 2)

## 7 Discussion

This qualitative study emphasises the importance of the relational work carried out by PC professionals in PC day hospital for patients with advanced cancer. The results allow us to determine the nature of this relationship, as well as its enablers and barriers, and the findings were similar in the two participating cancer centres. One of the main goals of the relational work is to educate patients and their family members about the untreatable status of their cancer, and the irreversible decline in their physical health. This awareness is a prerequisite for accepting the decision to stop cancer treatment. It can pave the way for discussions about end‐of‐life care, ensuring it aligns with the patient's and their loved ones' priorities. These sensitive discussions require intensive use of interactive tactics to initiate them and a repeated search for active engagement by the patient and family throughout the conversations.

Compared with outpatient PC consultation, this relational work is favoured by the specific care conditions at the PC day hospital. First, it is a place of care dedicated to and piloted by the PC team, which can provide gestures of hospitality. Second, repeated sessions with the same interlocutors in the PC team promotes the establishment of trust. Third, the immediate interactions during several hours between health care professionals enhance interdisciplinary team work as well as a shared communication strategy and decision‐making with the oncologist on cancer treatment discontinuation. Indeed, this relational work is quite feasible during hospitalisation in the PC unit. However, the PC day hospital enables this type of work to be started earlier in the course of the disease for outpatients who are still undergoing cancer treatment and who did not need any stay in a PC unit.

EOL discussions is an essential issue for PC teams at PD day hospital, whose main determinant may be its integration into the mental framework of the patient and their loved ones. Glaser and Strauss described four main mental contexts according what the patient's position and what he or she assumes that the other knows [29]: closed (the patient does not realize his imminent death although everyone knows), presumed (the patient suspects what others know and tries to confirm or dismiss the suspicion), of mutual pretence (each side considers the patient as dying but pretends that the other is unaware of it), opened (both medical staff and patient are aware that the patient is dying and speaks openly about it). Considering the theoretical framework proposed N. Dodier, a French sociologist, the relational work carried out by the PC team at PD day hospital could correspond alternatively to the clinical frame (diagnosis and therapeutic decision are driven by biomedical knowledge and is explained by the physician), the psychosomatic frame (the complaints are heard and interpreted in order to determine the way to act), the solicitude frame (physician listens to patient's psychological suffering which he considers his duty to appease) [31].

Previous qualitative studies have analysed healthcare professionals' points of view, identifying them as frequent barriers to EOL discussions [32, 33, 34]. In a Colombian study involving oncologists and palliative care specialists, physicians reported that many patients deny their imminent death, which hinders shared decision‐making and conversations [32]. They also mentioned frequent ambiguity regarding who should initiate conversations about end‐of‐life decisions with patients, and who should make the final decision. In another Japanese study, frequent barriers identified were patients' lack of understanding of the illness trajectory and collusion in doctor–patient communication about imminent death [33]. When analysing factors influencing the process of identifying patients for serious illness conversations, the PC team stated that continuity of relationships over time could facilitate this process [34].

### 7.1 Study Limitations

The study has several limitations that should be outlined. Firstly, a more relevant approach to addressing theobjective would have been to conduct non‐participative observations and analyse the recorded consultations, alongside PC healthcare interviews. Furthermore, caution should be exercised when considering the transferability of the findings to other countries due to differences in their healthcare systems and cultural patterns. Finally, the risk of contamination from one interview to the others with the same PC professional might have inserted bias into the data.

### 7.2 Clinical Implications

Findings illustrate that PC delivery during day hospitals integrated in cancer centres is characterised by the central role of relational work, with two main goals: raising patient and family awareness of the irreversible decline and approaching death and facilitating the timely decision to discontinue anti‐cancer treatment with a common communication strategy with the oncologist. This relational work is facilitated by some key conditions, which can be found in the context of day hospitals. These include extended consultation times, shared consultation time with the oncologist, and interdisciplinary work with supportive care professionals.

## 8 Conclusions

Ambulatory PC in the day hospital for advanced cancer patients could thus contribute to improving the quality of life of patients, improving their perception of the quality of care and reducing the aggressiveness of end‐of‐life care. We are conducting a multicentre randomized study in France to test this hypothesis. National government programmes for the development of early palliative care in oncology should promote the systematic creation of PC day hospitals in cancer centres.

## Author Contributions

J.C. M and E.L.: qualitative researchers. A.B., C.L., T.M., L.T. and E.G.: PC physicians at PC day hospital. J.C.M. and C.B.: interpretating the results, writing the manuscript. All authors: editing the manuscript.

## Ethics Statement

Ethics approval was obtained from Internal Institutional Review Board (CRI) and external Research Ethics Committee (CPP) (date: 2 March 2020, number: 2019‐A03116–5). This reserach followed the norm Consolidated criteria for reporting qualitative research (COREQ).

## Conflicts of Interest

The authors declare no conflicts of interest.
